# Notes and News

**Published:** 1952-10

**Authors:** 


### *Notes and News*

### EXAMINATION SUCCESSES

| | |
|---|---|
| *M.R.C.P. (London)* | Lisa E. Mandelbaum.<br>G. Walters. |
| *M.R.C.P. (Ed.)* | C. D. R. Pengelly. |
| *D.M.R.D. (R.C.S.)* | W. A. Copland.<br>D. C. R. Burrows.<br>I. R. S. Gordon.<br>C. Y. Lien. |
| *D.R.C.O.G.* | Luba Epsztejn. |
| *M.D. in State Medicine (Bristol)* | M. M. Lewis. |
| *M.D. (Aberdeen)* | Jean M. Sheach. |
| *D.P.M.* | W. L. Walker. |
| *F.R.C.S. (Ed.)* | E. M. Sparrow. |
| *L.R.C.P., M.R.C.S.* | R. B. Handscombe.<br>H. G. Gage. |

Dr. A. C. Fisher, M.D., F.R.C.S., has been awarded the O.B.E. in the New Year Honours for " medical services and welfare work in Northern Rhodesia ".

A gift to the Department of Dental Surgery of a collection of lantern and microscope slides, collected in Singapore, has been made by Professor E. K. Tratman, a former member of the Dental School Staff and a Bristol Graduate.

Dr. J. H. Middlemiss has been appointed as the University's representative at the Seventh International Congress of Radiology 1953 in Copenhagen.

The First World Conference on Medical Education is to be held at B.M.A. House, London, from August 24th to 25th, 1953. Details are available in the Dean's office.

The following prizes have been awarded:

*David Raynor Prize*—M. E. Jackman.
*Gold Medal for Child Health and Paediatrics*—N. G. Sanerkin.
*Attenborough Silver Medal*—K. J. Mullan.
*Attenborough Bronze Medal*—M. D. H. Allen.
*Russel Cooper Prize*—Marguerite E. Pennefather.
*Phillips Prize*—T. F. Harris.
*Bristol Royal Hospital Gold Medal*—F. J. Appelbe and N. J. Sanerkin (jointly).

The St. Andrew's Studentship in Neurophysiology and Psychiatry has been awarded to Mr. P. L. Short of the Burden Neurological Institute.

Mr. D. Gifford, B.Sc., of the Physics Department of Bristol General Hospital, has been given the status of Recognized Teacher in Medical Physics.

### " THE TEACHING OF ANAESTHESIA IN EUROPE "

The Editor regrets that in our annotation under this heading (July 1952) the name of Dr. JOHN H. CHALLENGER was inadvertently omitted as an instructor at the W.H.O. Anaesthesia Centre in Copenhagen.

### UNIVERSITY OF BRISTOL

*Degrees of M.B., Ch.B. (Brist.) December* 1952.

Barker, Kenneth James

Barton, Marjorie Edith

Blackwell, Noel Gerald

Bollen, Arthur Reginald

Booker, Ann Emily

Causton, John Clifford

Christie, Anne Winthrop

Claremont, Jean Margaret

Ginsberg, Barnett

Moore, Alan

Palmer, Ian William

Prichard, Mervyn Niblett

Sherwood, Peter Henry

Smith, Herbert Saviour

Stone, Derek William George

Tovey, John Ernest

Walker, Henry Aidan Patrick

### UNIVERSITY OF BRISTOL MEDICAL POSTGRADUATE DEPARTMENT

*Lecture Demonstrations*

A series of lecture demonstrations will be held in April/June 1953, intended for General Practitioners.

Sunday, April 12th: **Dr. G. E. F. Sutton, M.C.,** Southmead Hospital, 10.30—**Cardiac Emergencies.**

Sunday, April 19th: **Dr. J. M. Naish,** Southmead Hospital, 10.30 a.m.—**Anaemia.**

Thursday, April 23rd, and Friday, April 24th: Practical Demonstrations in Obstetrics, 8.15 p.m., Southmead Hospital.

Sunday, April 26th: **Dr. G. R. Airth,** Southmead Hospital, 10.30 a.m.—**X-rays.**

Sunday, May 3rd: **Dr. J. M. Naish,** Southmead Hospital, 10.30 a.m.—**Diabetes in General Practice.**

Sunday, May 10th: **Dr. J. M. Naish,** Southmead Hospital, 10.30 a.m.—**Dyspepsia.**

Saturday, May 16th, and Sunday, May 17th: Week-end course in Obstetrics.

Thursday, May 21st, and Friday, May 22nd: Practical Demonstrations in Obstetrics, 8.15 p.m., Southmead Hospital.

Wednesday, May 27th, and Thursday, May 28th: **Prof. J. M. Yoffey,** Exhibition, Department of Anatomy, University Road, 2—5 p.m.

Sunday, June 7th: **Dr. C. D. Evans, O.B.E.,** Bristol General Hospital, 10.30 a.m.—**Dermatology.**

Saturday, June 13th: **Dr. R. E. Hemphill** has arranged a demonstration and discussion on various psychiatric problems at Barrow Hospital, commencing at 2.30 p.m.

Sunday, June 14th: **Dr. R. P. Warin,** Bristol General Hospital, 10,30 a.m.—**Dermatology.**

Sunday, June 21st: **Dr. James Macrae,** Ham Green Hospital, 10.30 a.m.—**Antibiotics in Infectious Diseases.**

Sunday, June 28th: **Dr. James Macrae,** Ham Green Hospital, 10.30 a.m.—**Antibiotics in Infectious Diseases.**

*Inquiries should be addressed to Dr. A. H. Gale; Telephone* 24161.

### WESSEX RAHERE CLUB

The Spring Dinner of the above club will take place at the Castle Hotel, Taunton, on Saturday, April 18th.

It is hoped that, as usual, a member of the staff will be present as guest of honour.

Membership of the club is open to all Barts men practising in the West Country. Further details will be circulated to members and to any other Barts men who are interested and who will get in touch with the Hon. Secretary, Mr. A. Daunt Bateman of 3 The Circus, Bath.

